# In Utero Gene Therapy (IUGT) Using GLOBE Lentiviral Vector Phenotypically Corrects the Heterozygous Humanised Mouse Model and Its Progress Can Be Monitored Using MRI Techniques

**DOI:** 10.1038/s41598-019-48078-4

**Published:** 2019-08-12

**Authors:** Panicos Shangaris, Stavros P. Loukogeorgakis, Sindhu Subramaniam, Christina Flouri, Laurence H. Jackson, Wei Wang, Michael P. Blundell, Shanrun Liu, Simon Eaton, Nahla Bakhamis, Durrgah Latchumi Ramachandra, Panayiotis Maghsoudlou, Luca Urbani, Simon N. Waddington, Ayad Eddaoudi, Joy Archer, Michael N. Antoniou, Daniel J. Stuckey, Manfred Schmidt, Adrian J. Thrasher, Thomas M. Ryan, Paolo De Coppi, Anna L. David

**Affiliations:** 1 Institute for Women’s Health, University College London, 86-96 Chenies Mews, London, WC1E 6HX UK; 2 UCL Institute of Child Health, UCL, London, United Kingdom; 3 Biochemistry and Molecular Genetics, UAB, Birmingham, Alabama United States; 4 Department of Translational Oncology, National Centre for Tumour Diseases, Heidelberg, Germany; 5 Central Diagnostic Services, Queen’s Vet School Hospital, University of Cambridge, Cambridge, United Kingdom; 6 Centre for Advanced Biomedical Imaging, UCL, London, United Kingdom; 7 Wits/SAMRC Antiviral Gene Therapy Research Unit, Faculty of Health Sciences, University of the Witwatersrand, Johannesburg, South Africa; 8 Department of Medical and Molecular Genetics, KCL, London, United Kingdom

**Keywords:** Gene expression, Gene expression, Experimental models of disease, Translational research, Haematopoietic stem cells

## Abstract

In utero gene therapy (IUGT) to the fetal hematopoietic compartment could be used to treat congenital blood disorders such as β-thalassemia. A humanised mouse model of β-thalassemia was used, in which heterozygous animals are anaemic with splenomegaly and extramedullary hematopoiesis. Intrahepatic *in utero* injections of a β globin-expressing lentiviral vector (GLOBE), were performed in fetuses at E13.5 of gestation. We analysed animals at 12 and 32 weeks of age, for vector copy number in bone marrow, peripheral blood liver and spleen and we performed integration site analysis. Compared to noninjected heterozygous animals IUGT normalised blood haemoglobin levels and spleen weight. Integration site analysis showed polyclonality. The left ventricular ejection fraction measured using magnetic resonance imaging (MRI) in treated heterozygous animals was similar to that of normal non-β-thalassemic mice but significantly higher than untreated heterozygous thalassemia mice suggesting that IUGT ameliorated poor cardiac function. GLOBE LV-mediated IUGT normalised the haematological and anatomical phenotype in a heterozygous humanised model of β-thalassemia.

## Introduction

β-thalassemia is caused by mutations in the β-globin (*HBB*) locus giving β-globin chain reduction. It is a common severe disease with 56,000 β-thalassaemia major (TM) births per year worldwide and an estimated 5–7% carrier frequency in the general population, with increased frequency in risk ethnic groups such as Mediterranean, Middle Eastern, and South Asian^1^. TM requires regular blood transfusions and if left untreated is a life-threatening condition for which the only curative therapy - allogeneic haematopoietic stem cell (HSC) transplantation - is limited to a small percentage of patients with available HSC donors. It can be diagnosed *in utero*, and many countries have efficient screening and prenatal diagnosis programs^2^ that may lead to a termination of pregnancy (TOP) if an affected fetus is found. Prenatal diagnosis can detect an affected fetus as early as ten weeks of gestation^3^.

In TM, ineffective erythropoiesis results in expanded marrow cavities that impinge on healthy bone resulting in osteoporosis and osteonecrosis. These can distort the cranium, and of facial and long bones. Symptoms include erythropoiesis in extramedullary hematopoietic sites, hepatosplenomegaly, and, in some cases, extramedullary mass. Without transfusion support, 85% of patients with severe homozygous or compound heterozygous β-thalassemia will die by five years of age from severe anemia^4^. This is a problem in the countries where the cost or blood transfusion is prohibitively expensive and often unavailable.

Fetal therapy of an affected fetus would avoid termination of pregnancy, early death or life-threatening complications for patients with no compatible donor. This would have an impact where α-and β-thalassemia are most prevalent, even where termination of pregnancy may not be available, and blood transfusions are prohibitively expensive^5^.

Possible curative options that show promise are (i) the autologous correction of β-thalassaemia patient HSCs through gene augmentation studies mediated by β-globin expressing lentiviral vectors and (ii) reactivation of the endogenous *HBG* through lentiviral-mediated gene silencing of *HBG* silencer genes (e.g. *BCL11A*)^6^.

Various groups have been trying to construct an ideal gene delivery vehicle, which will be able to correct the disease either in utero or postnatally^7–11^. We choose the GLOBE^12,13^ lentiviral vector for an *in utero* therapy approach in a novel humanised model of β-thalassemia^14–17^. The GLOBE LV, which is now in phase I/II clinical trial (NCT02453477) was previously shown to correct β-thalassaemia in murine (Miccio *et al*.)^13^ and human (Roselli *et al*.)^12^ haematopoietic progenitor cells. In the study of Miccio *et al*., the authors showed an *in vivo* selection of genetically modified erythroblasts in β-thalassaemia mice^13^ with 15–30% of maturing erythroblasts being sufficient for correction and correction of human HP cells respectively. Recently A.A. Thompson and colleagues showed correction of transfusion-depended β-thalassaemia and reduction or elimination of transfusion requirements using *ex vivo* gene therapy with LentiGlobin BB305 vector^18^.

In this study we used the humanised mouse model of β-thalassemia which had been generated by replacing the mouse adult α- and β-globin genes with adult human α-globin genes and a human fetal to adult haemoglobin-switching cassette^16,17,19^. Two mouse models were used, one with functional HBB (B383: hBThal-Control) and one with non-functional HBB (B382: hBThal). The B382 with a human fetal to adult haemoglobin-switching cassette (non-functional *HBB*) kept in a homozygous human alpha globin and heterozygous human fetal to adult Hb-switching cassette. The homozygous animals from the later mouse strain die within two weeks if left untreated. This provides a similar temporal onset of the disease as in humans (early years) if left untreated. Animals become severely anaemic after birth and die within approximately 2–3 weeks of age, similar to the affected human neonate. However, animals can be rescued from lethal anaemia by weekly blood transfusions^20–22^. Heterozygous animals show features of thalassemia intermedia such as splenomegaly, extramedullary hematopoiesis, anaemia, and anisocytosis.

In this study, we show the phenotypic (haematological and anatomical) correction of the heterozygous animals using *in utero* gene therapy (IUGT) approach through prenatal intrahepatic injection of the GLOBE LV.

## Results

### In utero gene therapy corrects haemoglobin in a heterozygous humanised mouse model of thalassemia at 12 weeks’ post-IUGT

#### Results of surgery and postnatal outcome

In order to identify the true fetal and dam survival after in utero gene therapy delivery we analysed the fetuses at E18 after IUGT in two dams. In a representative dam, where all ten fetuses were injected, at post-mortem, a total of 3 fetuses were identified as alive, while the rest of the injected fetuses suffered with fetal demise. In another dam where all six fetuses were injected, three were identified as alive at E18. This gives a survival rate after injections of 37%. This was similar to the survival rate of 36% from IUGT to post-mortem analysis at either 12 or 32 weeks postnatally, suggesting that the loss was due to the *in utero* injection itself.

Examination of demised fetuses revealed that they probably died immediately after the injection since the fetuses were barely seen at the E18 time-point having been resorbed over the previous 4.5 days. Indeed, in some cases, only the placenta was visible when the dam was sacrificed. The fetuses which were collected at E18 were also genotyped to identify the presence of the homozygote, heterozygote and wild-type animals. The proportion of homozygous pups in uninjected litters at E18 was 25% from n = 3 dams.

Dam survival after IUGT was 100% (n = 9). All fetuses were injected in all litters n = 67 total injected; survival from IUGT to cross-fostering was n = 25. Overall pup survival to post-mortem analysis was 37% for all experiments. There was no difference in the weight of all groups at 12 Weeks (Untreated thalassaemia Control 28.79 ± 2.08 vs Humanised Control 28.09 ± 2.12 vs IUGT 28.74 ± 1.41, p = 0.82, One Way ANOVA).

The animals were cross-fostered to CD1 time mated dams which delivered one day earlier. This was done to avoid maternal cannibalism and also to prevent the passage of maternal antibody against the transgenic protein in the milk. Genotyping at three weeks after birth identified thalassaemia animals all of which were heterozygous. There were no homozygous pups identified by genotyping suggesting that they had demised either *in utero*, after the intrahepatic injection, or just before cross-fostering as all live cross-fostered pups survived to genotyping. Wild-type animals were excluded from further analysis once identified.

#### Phenotype results

**Figure 1:**
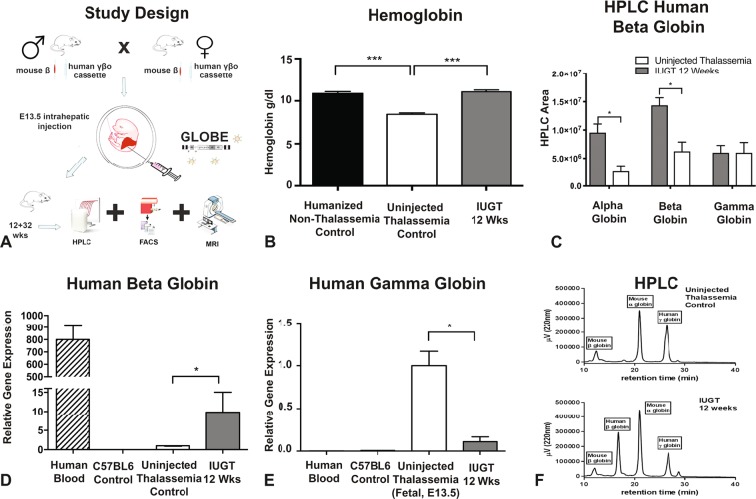
Study Design and Phenotypic Correction at 12 Weeks. (**A**) Study design: Heterozygous males were mated with heterozygous females. At E13.5 in utero gene therapy was performed by injecting 20 μl of the GLOBE vector into the intrahepatic space of each fetus in the litter after exposure of the uterus at laparotomy. The dams were allowed to litter, and the pups were cross-fostered into CD1 time time-mated dams to avoid maternal antibodies towards the virus. Post-mortem and analysis were performed at 12 weeks in the first study and 32 weeks in the second. Any wild-type animals were excluded from analysis. (**B**) Measurement of Hemoglobin in Humanised Non-Thalassemia Control 11 ±  0.21 Versus Uninjected Thalassemia Control 8.4 ± 0.17 Versus IUGT 12 Weeks 11 ± 0.22, n = 8, ***p < 0.0001, One Way ANOVA, Bonferroni’s Multiple comparisons test. (**C**) Quantification of High-Performance Liquid Chromatography showing an increase of both alpha and beta globins (p = 0.0044) with the gamma globin remaining stable and similar between the two groups. (**D**) Real Time PCR of human beta globin relative gene expression showing upregulation of the beta-globin gene in the IUGT treated animals compared to untreated thalassemia controls. Wild-type C57BL6 and human blood were used as positive and negative primer controls, n = 10, p = 0.039, ANOVA, Kruskal-Wallis test, Two-stage linear step up procedure of Benjamini, Krieger and Yekutieli. (**E**) Real Time PCR of human gamma-globin relative gene expression showing downregulation of the gamma-globin gene in the IUGT treated animals compared to untreated fetal thalassemia controls at E13.5, 0.11 ± 0.024 versus 1.0 ± 0, n = 10, p = 0.0002, ANOVA, Kruskal-Wallis test, Two-stage linear step up procedure of Benjamini, Krieger and Yekutieli. Wild-type C57BL6 and human blood were used as negative primer controls. (**F**) Representative figure of High-Performance Liquid Chromatography showing the presence of a human beta globin chain pick in the IUGT group.

At 12 weeks of age, the peripheral blood haemoglobin concentration of treated heterozygous pups was not significantly different to that of Humanised non-thalassemia control animals (11 ± 0.21 vs 11 ± 0.22, p > 0.99, One Way ANOVA, Fig. 1B), and was significantly higher than the haemoglobin concentration of untreated heterozygous control pups (8.4 ± 0.17, p = 0.0001). The relative expression of the human beta-globin gene in the peripheral blood confirmed that the normalised haemoglobin concentration was due to the increased expression of the transgene (n = 10, p = 0.039, One Way ANOVA, Kruskal-Wallis test, a Two-stage linear step-up procedure of Benjamini, Krieger and Yekutieli, Fig. 1D). At 12 weeks of age, the relative gene expression of human gamma-globin was lower in GLOBE, IUGT, treated compared to untreated heterozygous thalassemia pups (0.11 ± 0.024 versus 1.0 ± 0, n = 10, p = 0.0002, one-way ANOVA, Kruskal-Wallis test, a Two-stage linear step-up procedure of Benjamini, Krieger and Yekutieli, Fig. 1E). This confirmed that in treated heterozygous thalassemia pups, the human gamma to beta-globin switching cassette successfully switched human gamma-globin to beta and subsequently fetal to adult haemoglobin. i.e. there was no hereditary persistence of fetal haemoglobin which could have been responsible for the higher Hb. This production of transgenic beta-haemoglobin was also verified at a protein level using high-performance liquid chromatography (HPLC) where the presence of a human beta chain peak was seen in the treated heterozygous thalassemia pups 12 weeks after injection (Fig. 1F). Data from HPLC were quantified and show an increase in the expression of human beta-globin chain at a protein level (Fig. 1B). The alpha globin in the treated animals was also higher than untreated and the level of gamma-globin was not affected by IUGT.

**Figure 2:**
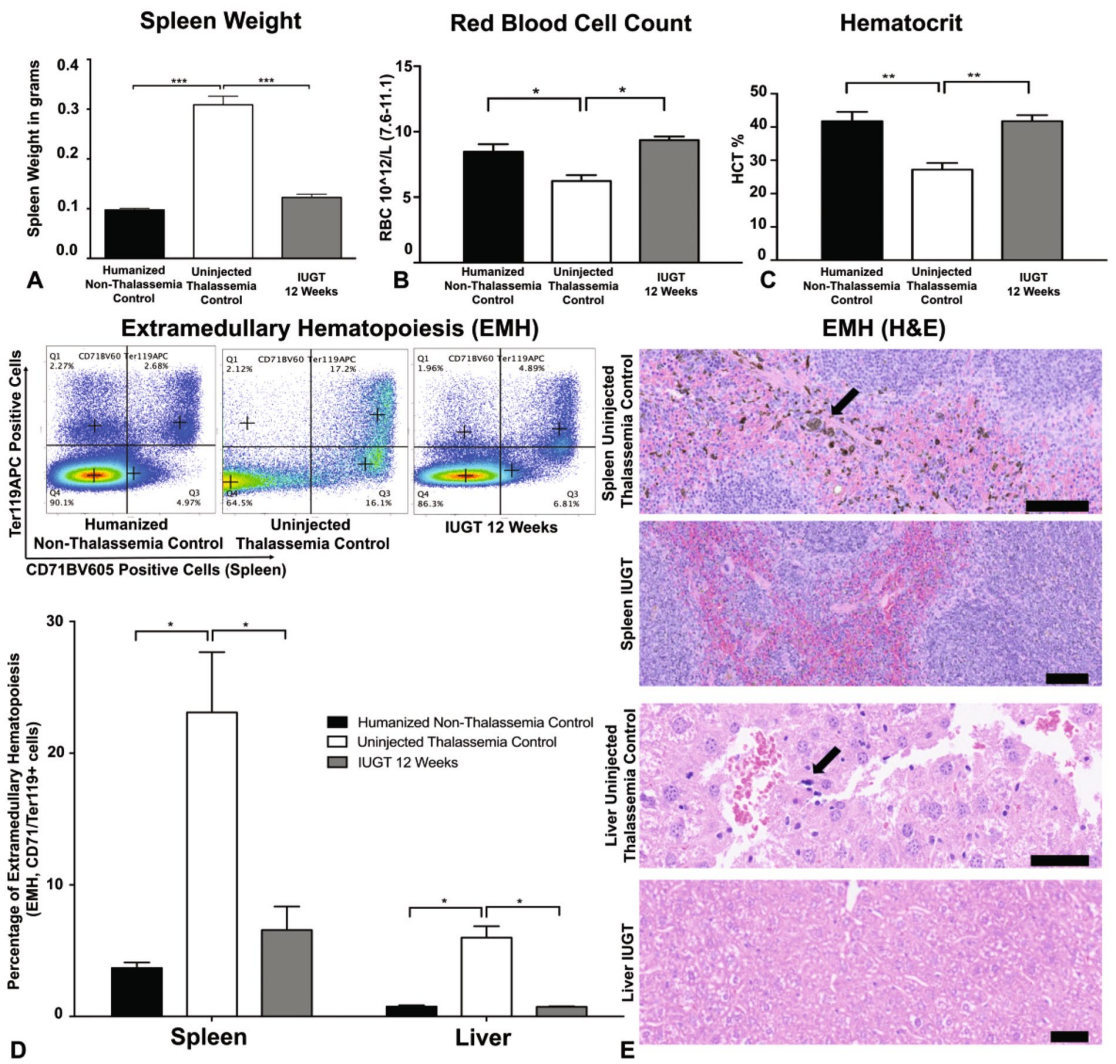
Correction of Hematological Indices and Extramedullary Hematopoiesis. (**A**) Spleen weight in IUGT 12 Weeks animals was significantly lower 0.12 ± 0.0065 than untreated thalassemia controls 0.31 ± 0.017, n = 8, p < 0.0001 and similar to Humanised Non-Thalassemia Control 0.098 ± 0.0031, One Way ANOVA, Bonferroni’s Multiple comparisons test. (**B**) Red Blood Cell Count in IUGT 12 Week animals was significantly higher 9.36 ± 0.27 10^12/L, n = 8 than untreated thalassemia controls 6.23 ± 0.45 10^12/L, n = 8, p = 0.0015 and similar to Humanised non-thalassemia controls 8.47 ± 0.57 10^12/L, n = 8, p = 0.089, One Way ANOVA, Bonferroni’s Multiple comparisons test. (**C**) The Hematocrit in IUGT 12 Week animals was significantly higher 41.79 ± 1.76%, n = 8, than untreated thalassemia controls 27.19 ± 2.02, n = 8, p = 0.0025 and similar to Humanised non-thalassemia controls 41.76 ± 2.80, n = 8, p > 0.99, One Way ANOVA, Bonferroni’s Multiple comparisons test. (**D**) Extramedullary Hematopoiesis in the Spleen was significantly lower in IUGT 12 Week animals 6.57 ± 1.78, n = 4, versus uninjected thalassemia controls 23.10 ± 4.57, n = 3, p = 0.00075 and similar to Humanised non-thalassemia controls 3.69 ± 0.41, n = 6, p = 0.64. Similarly, in the Liver, the level of EMH was lower in the IUGT 12 Week animals 0.74 ± 0.042, versus uninjected thalassemia controls 5.99 ± 0.87, n = 5, p = 0.10 and comparable to Humanised non-thalassemia controls 0.75 ± 0.091, n = 4, p > 0.99, One Way ANOVA, Bonferroni’s Multiple comparisons test. (**E**) Hematoxylin & Eosin light microscope images showing areas of extramedullary hematopoiesis (arrows) in the Spleen (Scale Bar 100 μm) and Liver (Scale Bar 50 μm).

Heterozygous humanised thalassemia animals show evidence of splenomegaly, extramedullary and abnormal full blood count indices. In IUGT 12 Weeks animals the spleen weight was significantly less (0.12 ± 0.0065) than non-treated animals (0.31 ± 0.017, n = 8, p < 0.0001, One Way ANOVA), and similar to Non-thalassemia control animal^20^ (Fig. 2A). The haematological indices of IUGT mice, Red Blood Cell Count (Fig. 2B) and Hematocrit (Fig. 2C) were also corrected at 12 weeks to the levels of the Humanised Non-Thalassemia Control animals. The level of extramedullary hematopoiesis (ineffective erythropoiesis), indicated by the ratio of erythroid progenitor cells (CD71+Ter119+) outside the bone marrow was also significantly lower in IUGT 12 Weeks animals (p = 0.00075, One Way ANOVA) compared to untreated thalassemia controls and not statistically different from Humanised Non-Thalassemia Controls (p = 0.1, One Way ANOVA)^21^ (Fig. 2D). The reduction of extramedullary hematopoiesis was also confirmed by H&E staining of spleen and liver slides (Fig. 2E). These results are comparable to previous studies done on adult thalassemia mice^10,12,22^.

### Use of MRI techniques to monitor disease progression in animals treated with IUGT

**Figure 3:**
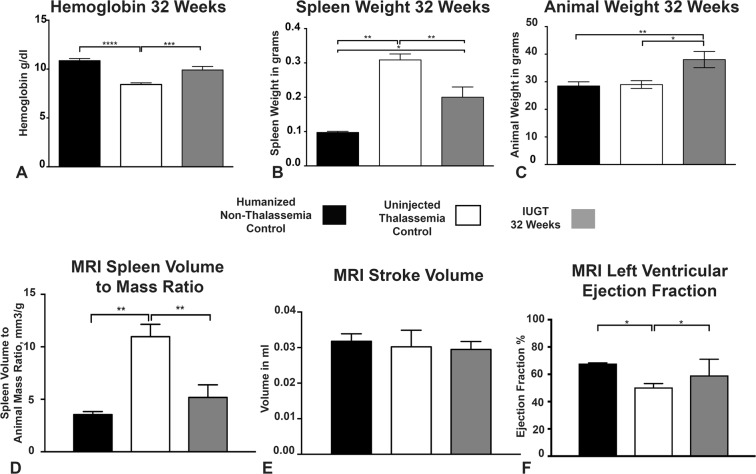
Long Term Correction and functional Assessment using MRI Techniques. (**A**) Hemoglobin levels in the IUGT 32 Weeks animals were significantly higher 9.91 ± 0.36, n = 8, than uninjected thalassemia controls 8.42 ± 0.167, n = 8 p = 0.0053 and similar to Humanised non-thalassemia control 10.87 ± 0.21, n = 8, p = 0.1, One Way ANOVA, Bonferroni’s Multiple comparisons test. (**B**) Spleen weight in the IUGT 32 Weeks animals was significantly lower 0.2 ± 0.03, n = 8 than uninjected thalassemia controls 0.309 ± 0.017, n = 8 p = 0.006 and similar but also significantly different to Humanised non-thalassemia control 0.1 ± 0.0031, n = 8, p = 0.01, One Way ANOVA, Bonferroni’s Multiple comparisons test. (**C**) Animal weight was similar in uninjected Thalassemia 28.97 ± 1.4, n = 9 and Humanised Non Thalassemia 28.46 ± 1.53, n = 8, p = 097, control animals, but significantly higher in IUGT 32 Weeks animals 38.03 ± 2.93, n = 6, p = 0.01, One Way ANOVA, Bonferroni’s Multiple comparisons test. (**D**) Spleen Volume to Animal Mass Ration (mm3/g) as measured by MRI was significantly lower in IUGT 32 Weeks animals 5.19 ± 1.20, n = 6 than Uninjected Thalassemia controls 10.97 ± 1.78, n = 6, p = 0.0012 and similar to Humanised non-thalassemia control 3.54 ± 0.28, p = 0.24, One Way ANOVA, Bonferroni’s Multiple comparisons test. (**E**) Stroke Volume (in ml) as measured by MRI was not significantly different in any of the groups at 32 weeks. IUGT 32 Weeks 0.029 ± 0.0022, n = 6 versus Uninjected Thalassemia control 0.03 ± 0.0047, n = 6, p = 0.987 versus Humanised Non-thalassemia control 0.032 ± 0.0021, n = 4, p = 0.902, One Way ANOVA, Bonferroni’s Multiple comparisons test. (**F**) Left Ventricular Ejection Fraction as measured by MRI (%) was significantly higher in the IUGT 32 Weeks group 59 ± 12, n = 6 than uninjected thalassemia control 50 ± 2.8, n = 6, p = 0125 and not significantly different from the Humanised non-thalassemia control 68 ± 0.41, n = 6, p = 0.91, One Way ANOVA, Bonferroni’s Multiple comparisons test.

In our second study, the in-utero gene transfer was performed as before, but the animals were sacrificed at 32 weeks of age having had MRI assessment two weeks before post-mortem. The animals were also genotyped, and wild-type animals were excluded from the analysis. The hemoglobin levels in the IUGT 32 Weeks group were also significantly higher (9.91 ± 0.36, n = 8) than that of the uninjected thalassemia control animals (8.42 ± 0.167, n = 8, p = 0.0053) but not significantly different from the humanised non-thalassemia control (10.87 ± 0.21, n = 8, p = 0.1, One Way ANOVA, Bonferroni’s Multiple comparisons test, Fig. 3A). The weight of the spleen was also significantly lower (0.2 ± 0.03, n = 8) than that of the uninjected group (0.309 ± 0.017, n = 8 p = 0.006), though significantly different from humanised non-thalassemia control group (0.1 ± 0.0031, n = 8, p = 0.01, Fig. 3B).

There was a difference in animal weight in the IUGT 32 Weeks group which was higher (38.03 ± 2.93, n = 6, p = 0.01, One Way ANOVA, Bonferroni’s Multiple comparisons test) than the uninjected Thalassaemia (28.97 ± 1.4, n = 9) and the age-matched humanised Non-Thalassaemia control (28.46 ± 1.53, n = 8) (Fig. 3C). This might be due to the viral integration to Socs6, which was observed during the site integration studies.

A preclinical MRI assessment was used to investigate, *in vivo*, the disease progress after in utero gene therapy as described^23^. Spleen volume was initially measured using MRI, and this was then calculated as an organ volume to mass ratio for a more accurate representation of organ volume.

**Figure 4:**
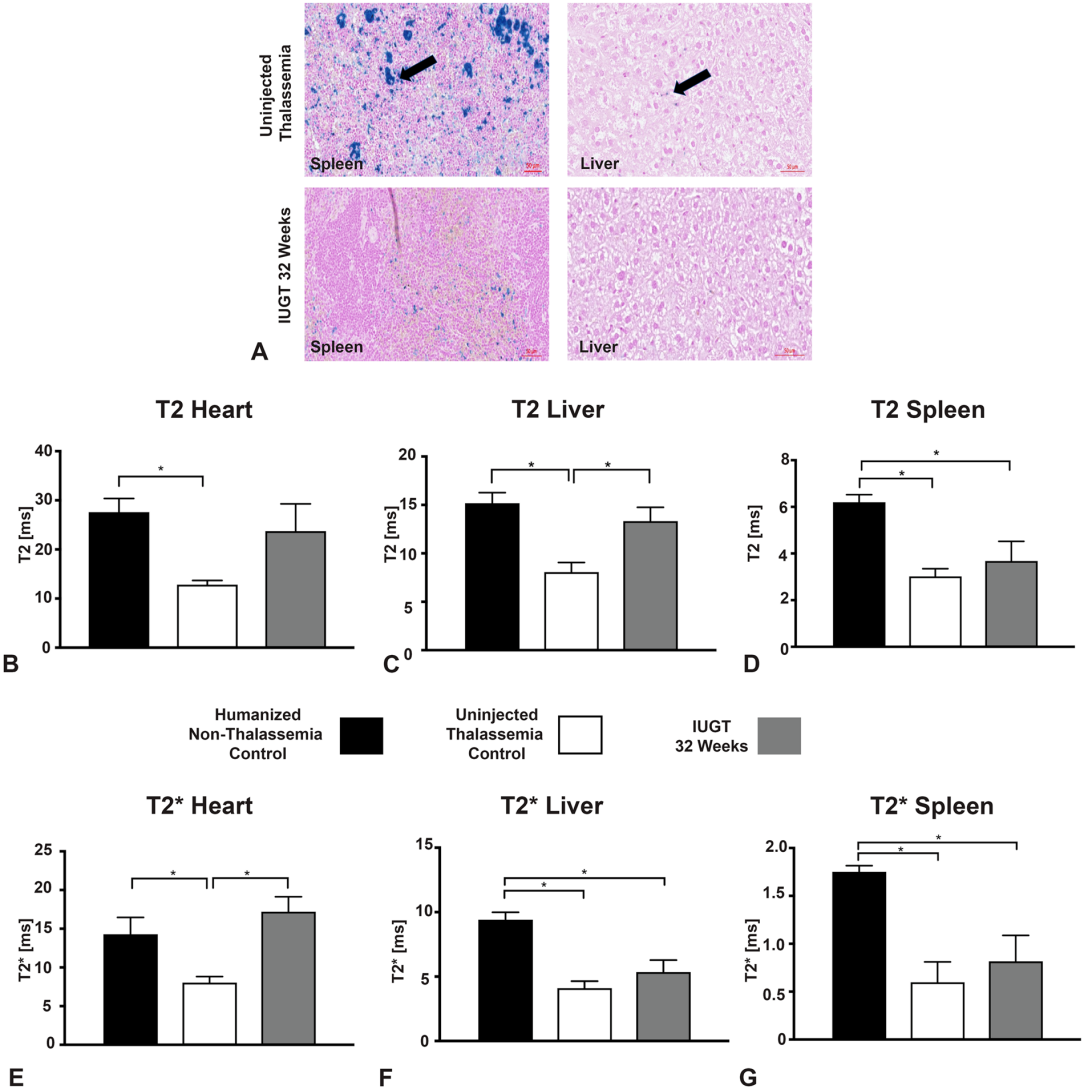
Iron Accumulation Assessment using MRI. (**A**) Representative Pearl Stained slides showing increased iron accumulation in the spleen and liver of the uninjected animals compared to IUGT 32 Weeks, (Scale Bar 50 μm). (**B**) T2 relaxation times for the IUGT 32 Weeks group, were not different in the Heart (p = 0.26–0.98, one-way ANOVA, Bonferroni’s Multiple comparisons test) compared to uninjected thalassemia control. (**C**) T2 relaxation times were different in Liver of the IUGT 32 Weeks group (13.5 ± 1.65 vs 8.068, n = 6, p = 0.0017) compared to uninjected thalassemia control and similar to Humanised non-thalassemia control (p = 0.52, one-way ANOVA, Bonferroni’s Multiple comparisons test). (**D**) T2 relaxation times in IUGT 32 Weeks group were not significantly different in Spleen (p = 0.41, one-way ANOVA, Bonferroni’s Multiple comparisons test) when compared to uninjected thalassemia control. (**E**) T2* relaxation times were different in the Heart of the IUGT 32 Weeks group (19 ± 1.40 vs 8.07 ± 0.77, n = 6, p = 0.0019) compared to uninjected thalassemia control and similar to Humanised non-thalassemia control (p = 0.32, one-way ANOVA, Bonferroni’s Multiple comparisons test). (**F**) T2* Relaxation Times in the Liver of IUGT 32 Weeks animals were significantly shortened 5.36 ± 0.91, n = 6 compared to Humanised Non-Thalassemia Control 9.34 ± 0.83, n = 6, p = 0.008 but not significantly different from the uninjected Thalassemia Control group, 4.76 ± 0.73, n = 6, p = 0.61, one-way ANOVA, Bonferroni’s Multiple comparisons test. (**G**) T2* in the spleen of IUGT 32 Weeks were significantly shortened 0.81 ± 0.27, n = 6 compared to Humanised Non-Thalassemia Control 1.72 ± 0.09, n = 6, p = 0.02 but not significantly different from the uninjected Thalassemia Control group, 0.60 ± 0.21, n = 6, p = 0.48, one-way ANOVA, Bonferroni’s Multiple comparisons test.

The Spleen Volume to Animal Mass Ration (mm^3^/g) was lower in IUGT 32 Weeks animals 5.19 ± 1.20, n = 7 than of the Uninjected Thalassaemia controls 10.97 ± 1.78, n = 7, **p = 0.015 and similar to Humanised non-thalassaemia control 3.54 ± 0.29, p = 0.24. This confirmed that the spleen volume was reduced, which was measured, without the need to sacrifice any of the animals (Fig. 3D). The heart stroke volume remained the same after IUGT (0.029 ± 0.0022ml, n = 6 versus Uninjected Thalassemia control 0.03 ± 0.0047ml, n = 6, p = 0.987 versus Humanised Non-thalassemia control 0.032 ± 0.0021ml, n = 4, p = 0.902, One Way ANOVA, Bonferroni’s Multiple comparisons test, Fig. 4E). This confirms that IUGT does not cause any structural defect, which was also confirmed on histopathological examination.

Encouraging results have arisen from the finding that the left ventricular ejection fraction (%) was increased in the IUGT 32 Weeks group (70.85 ± 1.58, n = 6) compared to the uninjected thalassaemia group (50.40 ± 2.83, n = 6, p = 0.0024) but was not different to the Humanised non-thalassaemia control group (68.00 ± 0.41, n = 6, p = 0.34, One Way ANOVA, Bonferroni’s Multiple comparisons test, Fig. 3F). We hypothesised that the iron accumulation in control non-injected group caused a reduction in the ejection fraction, which did not occur in the IUGT treated animals that had improved cardiac function^24–26^.

### Monitoring Iron accumulation in thalassemia animals using MRI T2* techniques

The IUGT 32 Weeks animals were scanned using MRI T2 and T2* relaxation times.

Tissue iron can be detected indirectly by measuring, using MRI, the relaxation times of hydrogen nuclei affected by ferritin and hemosiderin iron. The proton relaxations times are shortened as a result of the iron, specifically T2. The presence of blood can spoil the myocardium and blur its borders. This can be addressed by using T2* which excludes the signal from blood, using multiecho images in late diastole^27^.

T2 relaxation time was longer in IUGT 32 Weeks in the Heart 24 ± 5.5, n = 6 compared to Uninjected Thalassaemia Control 13 ± 0.83, n = 6, p = 0.26 and shorter, comparable to Humanised Non-Thalassaemia Control 28 ± 2.8, n = 6, p = 0.49. The T2 relaxation time of the untreated thalassaemia control was lower (p = 0.012) than the humanised non-thalassaemia control (Fig. 4B).

The T2 and T2* relaxation times in the spleen of the IUGT group were shortened compared to Humanised Non-Thalassaemia Control. No difference was seen between the IUGT group and the Thalassemia control, which was unexpected (Fig. 4D,G). This might be because of the increased breaking down of erythrocytes and subsequently iron accumulation in the spleens of the thalassaemia animals (IUGT and Thalassemia Control). The excess iron accumulation in the thalassaemia animals could be due to dietary iron, and excess accumulation could be specific to the animal model.

Even though the T2* in IUGT 32 Weeks was similar to uninjected Thalassemia Control group, representative Pearl stains from an animal with successful correction of the defect (HB:12.5g/dl) showed less iron pigmentation compared to an untreated thalassemia control (Hb:9.0g/dl) (Fig. 4A).

T2 relaxation times in the liver of IUGT 32 Weeks were similar to Non-Thalassaemia Control and different from the uninjected Thalassaemia Control group (Fig. 4C). This was not the case for the T2* relaxation times in the liver which were different to the Non-Thalassaemia Control and similar to the uninjected Thalassaemia Control group (Fig. 4F).

Non Invasive disease progress monitoring is possible with MRI, which corresponds to dry weight measurements^23^. For this experiment T2* was more accurate that T2 in the heart even through T2 had the same trend as T2*. This was also reported by L Jackson *et al*.^23^.

### Site Integration (SI) Studies in IUGT 12 Weeks Recipients

**Figure 5:**
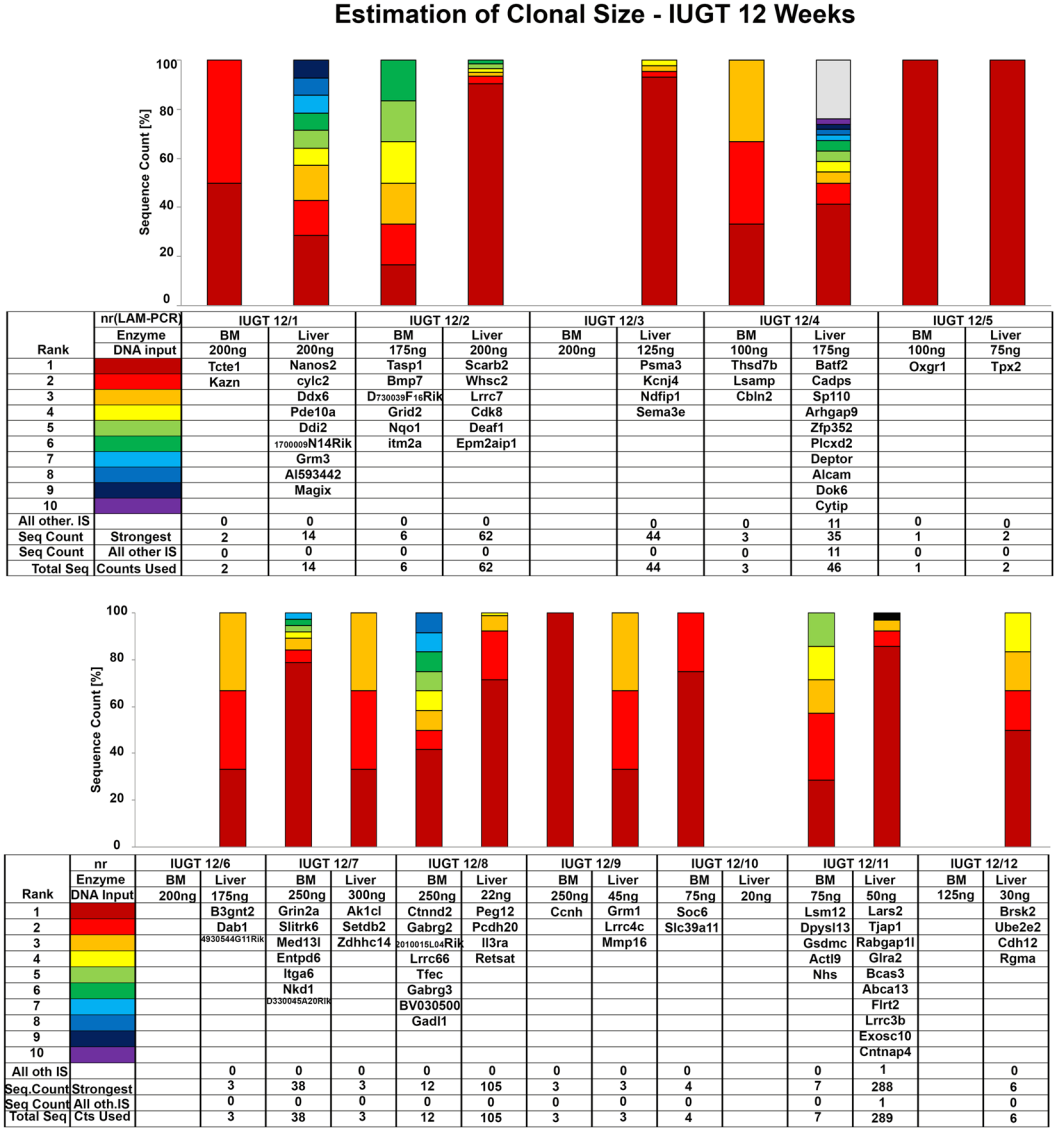
Site Integration Analysis. Vector integration studies were done in the animals which were sacrificed at 12 weeks. The integration site analysis was done on bone marrow and liver. No similar sites were observed.

**Figure 6:**
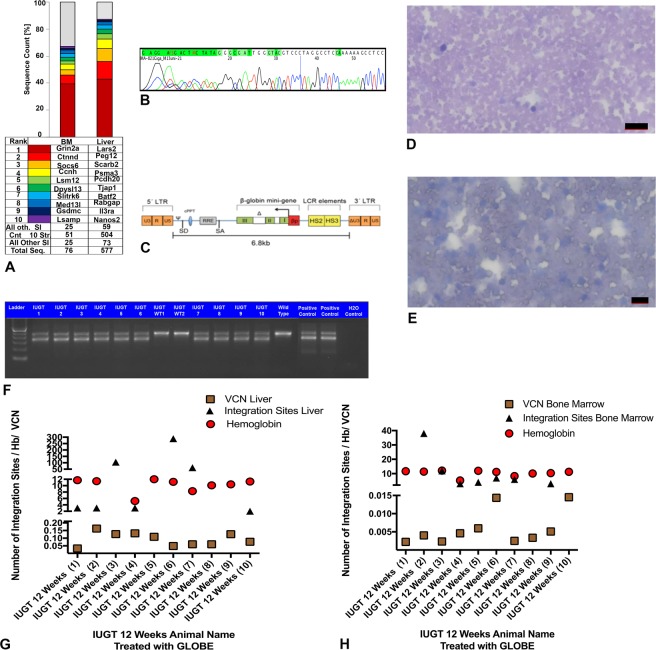
Vector Copy Number, Genotyping and Blood Film Analysis. (**A**) Common Integration sites in IUGT 12 Weeks animals. (**B**) Sequencing of the GLOBE plasmid. (**C**) Representative map of GLOBE Vector. (**D**) Representative blood film of mouse peripheral blood showing normal profile (Scale Bar 20 μm). (**E**) Representative Blood film of mouse blood from non-treated control thalassemia group showing moderate anisocytosis, polychromatic cells and a mixed population of normocytes, schistocytes, crenated cells and acanthocytes (Scale Bar 10 μm). (**F**) Genotyping of the mice showed heterozygosity for the human γβ^0^ switching cassette. Agarose gel electrophoresis of the PCR products of the IUGT, positive controls, and wild-type mice DNA. The mouse β-globin product is 429 bp in size, and the human γβ^0^ knocked-in product is 323 bp in size. Heterozygous mice exhibited both products. Homozygous mice showed only one product at 323 bp (not shown here). All the treated mice were heterozygous except for WT1 and WT2 which showed only the mouse β-globin product similar to the wild type. None of the treated mice were homozygous for the human γβ^0^ product. (**G**) Liver VCN (0.093 ± 0.14, n = 10) of individual animals in IUGT 12 Weeks with corresponding Liver Integration sites (60.56 ± 30.73, n = 10) and hemoglobin levels 11.22 ± 0.21. (**H**) Bone Marrow VCN (9.25 ± 4.27, n = 10) of individual animals in IUGT 12 Weeks with corresponding Bone Marrow Integration sites (0.0059 ± 0.0014) and hemoglobin levels (11.22 ± 0.21).

Vector integration studies were done in the animals which were sacrificed at 12 weeks. The integration site analysis was done on bone marrow and liver. Vector Integration Studies in IUGT 12 Weeks animals showed no similar integration sites (Fig. 5). The mean vector copy number (VCN) in the IUGT 12 Weeks group, in the liver, was 0.093 ± 0.14, n = 10 and the corresponding integration sites were 60.56 ± 30.73 (Fig. 6G). The VCN in the bone marrow was 0.0059 ± 0.0014, n = 10 and the corresponding integration sites were 9.25 ± 4.27 (Fig. 6H). No similar integration sites were found during the vector integration site analysis. Cumulatively the number of integration sites in the liver was 504 and in the bone marrow 51. The SI study highlighted the importance of investigating the specific vector in each biological application (IUGT) before human application. Even though oncogenesis through viral genome integration is intrinsically less likely in terminally differentiated cells, this is not the case for rapidly dividing cells of the haematopoietic lineage^28^. Themis and colleagues found a high incidence of hepatocellular carcinoma in mice receiving intravenous vitelline vein injection of the Equine Immune Anaemia Virus (EIAV) SMART 2hFIX, SMART 2Z, and SMART 3NZ lentiviral vectors *in* utero, but no oncogenesis was observed in HIV-1 based vector injections using the same route^29^. Thus the particular vector type may be of importance in the development of oncogenesis. Further studies suggest that the fetal mouse may be a sensitive genotoxicity model that exposes particular lentiviral-associated mutagenesis resulting in liver oncogenesis^30^. In this study, we observed integration to Peg12 gene which is associated with tumorigenesis but no hepatocellular carcinomas were observed at any time point. We did observe that IUGT animals were significantly heavier than non-thalassemic controls at 32 weeks. A possible explanation is integration to the gene SOCS-6, which was observed during the site integration studies. Socs-6 is involved in IGF-1 signalling and integration to the its site might have caused overexpression of the gene resulting to a significant weight difference^31,32^. Also, SI was observed in genes which are related to the development and function of the nervous system and also spermatogenesis. A future repeat study should include an assessment of the neural development of the offspring and also their reproductive potential, especially in males.

Vectors need to be assessed for safety and shown to have low oncogenic potential, in the actual biological system since the integration profile of the same vector may differ in different cell types, *ex vivo* and *in vivo* and during different developmental stages, before gene therapy application^33^. The safety in such system needs to be addressed^34^.

## Discussion

In this study, we show successful phenotypic correction of thalassaemia intermedia in a humanised mouse model using an *in*-*utero* gene therapy approach. Normalisation of haemoglobin, spleen size and a reduction in the level of extramedullary haematopoiesis were the main findings of this preclinical study. We proved that there was a successful fetal to adult haemoglobin switch in the treated mice, as this was necessary to demonstrate that the improved phenotype was due to the IUGT. *In vivo* mouse, MRI was used to monitor the disease^23^ and showed improved cardiac function in the IUGT treated pups, even at 32 weeks after birth. We hypothesise that this was mediated through reduced iron accumulation in the heart.

Fetal application of gene therapy in mouse models of congenital disease such as haemophilia A^35^ and B^36^, congenital blindness^37^, Crigler-Najjar type 1 syndrome^38^ and Pompe disease (glycogen storage disease type II)^35–39^ have previously shown phenotypic correction of the condition. Fetal gene therapy has also been found to prevent the development of fetal structural anomalies. For example transient transduction of the periderm via intra-amniotic delivery of adenoviral vector encoding transforming growth factor β3 (TGFβ3) prevents cleft palate in a mouse model of disease^40^. For obstetric conditions that affect the fetus, maternal uterine artery injection of adenovirus containing the vascular endothelial growth factor (VEGF) gene improves fetal growth in growth restricted sheep and guinea pigs^41,42^. With regard to thalassaemia specifically a study by May *et al*.,^43^ showed that gene transfer using a LV that contained proximal and distal regulatory elements with a large segment of the LCR (TNS9) could correct β-thalassaemia in mice, with an average increase in Hb by 3–4g/dL per vector copy and improvement of haematological indices. Similar studies have been published using other thalassaemia mouse models but with variable results^8,43–46^.

The target cell niche in this study was the expanding population of fetal liver haematopoietic stem cells. There are intrinsic differences between fetal and adult HSCs properties especially fetal liver HSCs which can give rise to thymocytes, especially Tregs which are responsible for promoting tolerance to self and potentially foreign antigen^47^. The fetal to adult switch in these HSC properties occurs between week one and two after birth in mice. During fetal life, the HSC pools in the fetal liver are actively cycling and rapidly expanding whereas, once the HSCs reach the fetal bone marrow, they become quiescent. This property of fetal HSC pools in the liver and their potential of thymic repopulation may be an added advantage for intrahepatic IUGT^48^ which is easily able to direct gene transfer to the fetal liver through direct injection. Fetal liver HSCs are also capable of repopulating five times more effectively long term than short term^49^. Any manipulation of liver HSCs should have a long-term effect, ideally permanent, especially if these cells migrate to the thymus^47^. Intrahepatic fetal gene transfer to haematopoietic progenitors has also been demonstrated in primates where the maximum effect was achieved using lentiviral vectors^50^.

Correction of haemoglobin was achieved with a low VCN of 0.093 in the liver. This is similar to the findings of Miccio *et al*.^13^ where correction of haemoglobin was achieved with an HSC VCN of <1 (0.2–0.9)^13^ in adult mice using *ex vivo* transduction of CD34+ HSCs before reinfusion in an autologous setting. The liver VCN in this study is 5–10 times lower than the HSC VCN that Miccio *et al*. observed^13^. The VCN in the liver was expressed in cells from the whole liver cell population and not only on the HSCs. There is a marked discrepancy between the VCN in the BM and that in the liver. This can be explained by the fact, that possibly, a large percentage of non-hematopoietic (off-target) cells were transduced following the direct intrahepatic lentiviral injection. Other authors have demonstrated that non-haematopoietic cells are transduced in an intrahepatic IUGT. The developmental stage of each organ, in this case the liver, could determine the susceptibility to in utero gene transfer and transgene expression^51^. The efficiency and distribution of transduction, is also highly depended on the type of vector used, as well as, the route of administration^52^. This could raise safety concerns, even though in a Cynomolgus Macaque model no tumours were observed, 6 years post IUGT, with an Adeno Associated viral vector. The authors, in this non-human primate study, addressed the importance for life long surveillance^53^. Thus, the VCN in the HSCs residing in the liver will be even lower. The use of direct *in utero* gene therapy, took advantage of the high proliferation rate of the fetal HSCs which resulted in an adequate number of transduced daughter cells that were able to correct the phenotypic Hb defect. It was not possible to do individual sorting of HSCs from each transduced mouse due to the minimal amount of tissue collected and the scope of analysis initially planned such as EMH, histology and HPLC. Thus, we were unable to calculate the VCN in the HSC population only and instead present data on the VCN for the whole heterogeneous population of hepatocytes and whole bone marrow. It was unexpected that a level of gene correction (VCN) in the BM of 0.5% resulted in such a substantial boost in hemoglobin levels in the periphery. It is possible that the HSCs might have had a greater percentage of initial correction since the fetal liver is mainly made of HSC progenitors. Alternatively this result may not be true as we concentrated on analysis of the whole bone marrow rather than just the BM HSCs and our VCN result may have been lowered due to dilution. Further studies are needed to confirm this effect. Previous studies have found low genotoxicity of lentiviral vector integration in mice that are prone to developing tumours^33^. Nevertheless, it is an important safety consideration to keep the VCN as low as possible whilst maintaining the efficacy of the gene transfer.

The humanised mouse model of beta thalassaemia^14,15,17,19^ that we used is unique in that the fetal mice produce human gamma-globin (human fetal haemoglobin) in utero^15^, and have a human gamma to beta switching cassette which results in the death of homozygous animals within two weeks of birth, as the production of defective beta globin takes over. Homozygous animals can be rescued postnatally using regular blood transfusions^16^. This model allows human beta-globin producing vectors to be tested *in utero*^12,13,54,55^, and since the *in utero* phenotype is less severe than of other thalassaemia mouse models, eg *th3*, it allows the evaluation of IUGT to rescue the mouse model *in utero* and post-natally^45,56^.

In the first cohort (12 Weeks), where the animals were analysed at 12 weeks after birth, we observed normalisation of the phenotype with functional correction of anaemia, excessive extramedullary haematopoiesis and the other abnormal haematological indices. Blood films and H&E were also examined by an expert (University of Cambridge, Veterinary College). There was no evidence of malignancy and no abnormal looking red blood cells in the corrected (Hb > 10.0g/dl) animals (Fig. 6D). On the contrary in the untreated animals (Hb < 9.0g/dl), we observed the presence of moderate anisocytosis, polychromatic cells and a mixed population of normocytes, schistocytes, crenated cells and acanthocytes^21^ (Fig. 6E).The presence of normal blood films in the treated animals compared to untreated was a confirmatory result. To confirm the presence of normal Hb we used real-time PCR and high-performance liquid chromatography to demonstrate beta-globin gene expression with downregulation of the gamma-globin gene as well as the additional peak from the beta globin chain in HPLC. These results excluded the hereditary persistence of fetal haemoglobin which could have contributed to the phenotypic correction of the overall haemoglobin.

In the long term 32-week postnatal group, the phenotypic correction appeared to be less effective as in the group analysed at 12 weeks after birth suggesting that the effect may reduce over time. This could be due to transcriptional silencing of the transgene^57^ or even due to epigenetic changes leading to deactivation of the beta-globin gene as the pups grew^58,59^. This was evident in the lower Hb concentration at the later time point (9.91 ± 0.36g/dl at 32 weeks’ vs 11 ±  0.22g/dl at 12 weeks), although the haemoglobin concentration at 32 weeks post-delivery was still significantly higher than that of age-matched controls. Histological analysis of the animals showed a correlation of spleen hyperplasia with iron deposits in the spleen, moderate anisocytosis and anaemia.

In the long-term group, MRI was used as a novel method of monitoring *in utero* gene therapy functional success (left ventricular ejection fraction, spleen volume, iron overload)^60–63^. We found that MRI can be used to monitor the disease progress, using T2 or T2* without sacrificing the animal. T2* is currently used clinically for the non-invasive diagnosis of iron overload in both heart and liver. It is also used for the assessment of the thalassemia major patient prior to stem cell transplantation^64^. This study confirms that this can be applied in small animal studies which can be used for the non-invasive assessment of new therapies^23^.

We did not observe any rescued homozygous pups in the genotyped litters; indeed, given the dam numbers and from previous studies in this specific humanised mouse model, we would have expected to have at least 5–6 (25% of the total) homozygous pups if they were corrected. The only homozygote pup had to be culled at 14 days of age, due to severe illness, and haemoglobin measurement showed severe anaemia (3g/dL). All live cross-fostered pups survived to genotyping. There are a number of possible reasons for this low survival of homozygote injected pups. It is possible that the treated homozygous pups demised *in utero* after the intrahepatic surgery or in the first hours after birth before they were cross-fostered due to a higher susceptibility to miscarriage or perinatal loss. Pups were usually born overnight and immediate cross-fostering was not possible as there was not always staff available to transfer them to the foster mother. It is also possible that homozygote rescue was incomplete rendering the born homozygote newborn pups more susceptible to being rejected or attacked and eaten by their mother. This has been reported in other studies where fetal intervention occurred^65^. Future studies using a higher titre of the vector will be conducted to determine whether this improves survival in the homozygous pups.

In this study, we show that targeting the specific stem cell niche^66,67^ early enough in gestation with gene therapy could be critical for fetal gene transfer, especially if most of the haematopoietic stem cells are within the compartment, in this case, the liver. Alternative routes such as intraperitoneal and intravascular injection need investigation to achieve a phenotypic cure, especially rescuing the homozygous animals. Boelig *et al*. found that the intravenous route was most successful for achieving rapid diffuse donor cell population of the fetal liver after in utero hematopoietic cell transplantation in mice^68^. Whether this achieves the same effect for fetal gene therapy remains to be seen. Importantly for clinical translation, the tissues were studied by an expert, and no animals were found to have malignancy, which is in keeping with other studies using this stable GLOBE vector^13,18^. This can also be confirmed that GLOBE vector is currently used in Phase II clinical trial, using autologous stem cell/gene therapy approach in adult patients^69^. The number of vector integration sites corresponded to the vector copy number, even though this was low. This could be a sign that if higher VCN is achieved, the level of haemoglobin could also rise.

The integration site analysis revealed integration to be associated with the neural system, stem cells, spermatogenesis and also with an oncogene. This must be carefully studied since the potential of tumor formation was shown to be high in a fetal context when using certain lentiviral vectors^29^. This also supports the use of an *ex*-*vivo* gene therapy approach using amniotic fluid stem cells or other highly efficient HSC progenitors^70^.

One of the benefits of perinatal treatment lies in the potential to limit organ damage through early intervention. Also, applying a therapy to the fetus, where stem cell proliferation is high, results in a higher number of transduced cells, which leads to a better therapeutic effect^71^. The fetus also offers a size advantage, allowing a higher vector-to-target cell ratio. Certain organs that are challenging to target after birth may be more easily accessible in the fetus due to their developmental stages or relative immaturity^72–75^. The fetal epidermis is an example of this, as it undergoes remodelling by programmed cell death and is replaced by mature keratinocytes. The epidermis forms a thick barrier to gene transfer following birth^76^ but could be better targeted *in utero*^77^.

One of the main obstacles of postnatal gene therapy is the development of an immune response against the transgenic protein or the vector^78^. This is of particular importance when gene therapy aims to correct a genetic disease, which completely lacks a gene product. It is also possible that some patients may have pre-existing antibodies to the viral vector that will inhibit long-term expression of the transgenic protein; this will limit therapeutic efficacy and prevent repeated vector administration. For example, pre-existing neutralising antibodies against adeno-associated virus (AAV) serotype 2 have been shown to interfere with AAV2 vector-mediated factor IX (FIX) gene transfer to the liver^78–81^. Delivering foreign protein to the fetus can provide an advantage of immune tolerance during fetal life, a concept first suggested more than 60 years ago^82,83^. Induction of tolerance relies on the introduction and expression of the foreign protein early in gestation before the immune system is fully developed. The protein needs to remain at a detectable level within the fetus and presented to the thymus at the correct time^84–88^. In a postnatal treatment this can be achieved by ex-vivo correction of autologous HSCs, which will prevent an immune response, since the immune system will recognise the autologous, corrected cells as self. This can be demonstrated in a study, by A Thompson and colleagues. Using the BB305 vector, it reduced or eliminated the requirement for blood transfusions in 22 patients with severe β-thalassemia. No complications or reactions to the vector were reported^18^.

IUGT was delivered at embryonic day (E13.5) just before the time of HSC fetal bone marrow colonisation in mice, which occurs around E16-E17^89^, and which corresponds to an equivalent of 10 to 12 weeks of gestation in a human fetus^90^. Fetal gene transfer to the haematopoietic compartment^91^ may offer a third option to couples with a prenatal diagnosis of a fetal congenital blood disorder, especially in the beta haemoglobinopathies^92^. For human therapy, non-invasive prenatal diagnostic techniques are being developed using maternal blood to diagnose fetal thalassaemia from 8–10 weeks of gestation^93^. This would allow enough time to deliver gene therapy to the human fetus using ultrasound-guided intrahepatic injection. Using this methodology, no *ex*-*vivo* expansion and correction of the appropriate haematopoietic progenitors is required. More animal studies are required with the aim to rescue the homozygous mouse model. If this is successful, treatment could even be applied in low-income countries where the palliative cost of homozygous patients is unsustainable for the local health systems^94–96^. In order to reach the clinic, a gradual progression from small animal disease models, such as the mouse, followed by studies in larger animals, for example sheep to test the feasibility and safety of the delivery method will be necessary. A reproductive toxicology study would then be required, which might be performed either in rabbits or possibly in non-human primates before establishing a fist-in-human clinical trial of fetal gene therapy^73,97^.

## Methods

### Animals, animal care, and procedures

Humanised CA mice (α2α1/α2α1, γ(HPFH117) β^0^/+, Heterozygous Thalassemia) and the HbA mice (α2α1/α2α1, hγβ^A^/hγβ^A^, Humanised Non-Thalassemia Controls) were obtained from Dr Thomas Ryan, the University of Alabama at Birmingham U.S.A.^15,19^. Experimental protocols were performed following the U.K Home Office Regulations and Guidelines for the Operations of Animals (Scientific Procedures) Act (1986) and approved by the local UCL animal welfare ethics committee. Mice were housed in single cages after plugging. The dams were given wet food after the laparotomy and closely monitored for the first seven days after the procedure. The animal experiments were done according to the project license PPL No: 70/7408 and Animals [Scientific Procedures] Act 1986 and the NC3Rs ARRIVE guidelines 2013. The operator was personal licensee PIL 70/23796.

### Lentiviral vector production

GLOBE Lentiviral vectors were produced as described using standard methods^13,98^. Compared to other transcription designs, GLOBE vector has only HS2 and HS3 which have the crucial LCR enhancer and chromatin-opening functions^99,100^. GLOBE-LV is a minimal LCR-β-globin transcription unit containing a 2.7 kb fragment encompassing LCR elements HS2 and HS3, linked to a fully functional mini-β-globin gene with 265 bp of 5′ and 300 bp of 3′ flanking sequences; intron 2 features an internal deletion, reducing its size to 257 bp.

### Uninjected control litters

Genotyping of control litters (n = 3) was performed at E18 to determine the proportion of heterozygous and homozygous pups.

### In utero injections

IUGT was performed in timed-mated pregnant heterozygous thalassemia mice as described^38,101^ with minor modifications. On E13.5 day of gestation, the animals were shaved in the abdominal area; the skin was cleaned using ChloraPrep® and under 5% isoflurane (VetTech Solutions Ltd, UK) general anaesthesia, a midline laparotomy was performed, and the uterine horns exposed under sterile conditions. 20 μl (10^7^ VP/ml) of Globe LV viral particles were injected through the myometrium into the hepatic region of each fetus using a 33-gauge needle (Hamilton, Switzerland). The uterus was returned to the maternal peritoneal cavity, the abdomen was closed using an absorbable vicryl 6-0 suture (Ethicon Inc., USA) and analgesia (Marcain, 0.25% AstraZeneca) was administrated. Mice were recovered in a warm cage (28 °C) overnight. After spontaneous delivery at E20-E21 day of gestation, treated pups were cross-fostered to CD1 dams by day 2–3 after birth, to avoid maternal cannibalism and maternal antibody response to the transgenic protein^102–104^. Pups were genotyped at three weeks of age; earlier genotyping was precluded by the Home Office Project license. The pups were bred until scheduled post-mortem examination at 12 and 32 weeks postnatal.

### Post-mortem tissue harvest

At post-mortem examination, blood (0.5–1 ml) was collected by cardiac puncture under terminal anaesthesia, induced by injecting 0.8 ml of 1.25% Tribromoethanol (Avertin Sigma- Aldrich, UK) solution. The spleen and liver were placed in 1.5 ml Safe-Lock microtubes (Eppendorf, UK). For bone marrow collection, the femur and tibia were separated from the body and muscle was removed off the bones. The epiphysis was removed from both ends and placed in 1.5 ml Safe-Lock microtubes (Eppendorf, UK). All the tissues were snap frozen in liquid nitrogen and stored at −80 °C.

### Isolation of bone marrow

The bone marrow was harvested by inserting a syringe needle (Terumo, myjector 27G, 0.4 x 12 mm, Belgium) into one end of the bone and flushing 1 ml of Phosphate Buffer Saline (1x PBS, pH 7.2) (Sigma, Life Sciences, USA) repeatedly through the bone. The bone marrow aspirates were collected into 1.5 ml Eppendorf tubes and centrifuged at 300xg for 5 minutes to pellet the cells.

### DNA Isolation and quantitation

DNA was isolated from bone marrow aspirates using Invitrogen PureLink Genomic DNA Mini kit (Cat No: K1820-01) as per manufacturer’s instructions. The concentration of the DNA samples was by the NanoDrop 1000 Spectrophotometer (Thermo Scientific, UK). The quality of the DNA samples was determined by calculating the A260/A280 ratio.

### PCR for genotyping

Animals were genotyped using primers specific for mouse or human haemoglobin (Table 1). The reactions were done in 0.2ml polypropylene PCR tubes (Corning) in a Thermo Scientific thermal cycler using MyRedTaq PCR mix (Bioline).

**Table 1:** List of Primers used for genotyping and RT-qPCR.

Gene	Primers/probe	Melting (˚C) Temperature
LRT Forward	TGT GTG CCC GTC TGT TGT GT	59.4
LRT Reverse	GAG TCC TGC GTC GAG AGA GC	63.5
LRT Probe	FAM -CAG TGG CGC CCG AAC AGG GA- TAM	65.5
mTitin forward	AAA ACG AGC AGT GAC GTG AGC	59.8
mTitin reverse	TTC AGT CAT GCT GCT AGC GC	59.4
mouse beta forward	GCT GCT GGT TGT CTA CCC TTG	61.8
human gamma forward	GTG GAA GAT GCT GGA GGA GAA	60.3
human beta forward	CGT GCT GGT CTG TGT GCT G	61
mouse alpha forward	AGC TGA AGC CCT GGA AAG GAT	59.8
human alpha forward	CAG ACT CAG AGA GAA CCC ACC AT	62.4
mouse beta reverse	CCC ATG ATA GCA GAG GCA GAG	61.8
Human gamma reverse	TGC CAA AGC TGT CAA AGA ACC T	58.4
human beta reverse	CTT GTG GGC CAG GGC ATT AG	61.4
mouse alpha reverse	GCC GTG GCT TAC ATC AAA GTG	59.8
human alpha reverse	GCC TCC GCA CCA TAC TCG	60.5
mouse beta probe	FAM -CCA GCG GTA CTT TGA TAG CTT TGG AGA CC –TAM	68.1
human gamma probe	FAM - AGG CTC CTG GTT GTC TAC CCA TGG ACC -TAM	69.5
human beta probe	FAM - CCC ATC ACT TTG GCA AAG AAT TCA CCC -TAM	65
mouse alpha probe	FAM -TGC TAG CTT CCC CAC CAC CAA GAC C -TAM	67.9
human alpha probe	FAM -TGC TGT CTC CTG CCG ACA AGA CCA A -TAM	66.3
mTitin probe	FAM - TGC ACG GAA GCG TCT CGT CTC AGT C -TAM	67.9
Mouse β KI reverse	GTCAGAAGCAAATGTGAGGAGCA	62.5
β KI forward	TTGAGCAATGTGGACAGAGAAGG	62.4
B383 KI reverse	GTTTAGCCAGGGACCGTTTCA	62

### Agarose gel electrophoresis

DNA samples were resolved by agarose gel (2%) electrophoresis in 1X Tris-Borate-EDTA (TBE) buffer. The agarose gel (2%) was made using 1XTBE plus SYBR DNA stain (1 in 10,000 dilutions, Sigma-Aldrich). 1ul of DNA was loaded onto the gel and fragments resolved at 140V, 90mA for 45minutes. Nucleic acid fragments were visualised on a UV transilluminator (UVP®, USA) and photographed under ultraviolet light employing the Bio-Doc-ItTM System (UVP®).

### RNA isolation and quantitation

RNA was extracted from blood and bone marrow samples of mice using the Bioline Isolate II RNA Mini kit (BIO-52072) manufacturer’s instructions. RNA from the human samples was extracted using TRIzol reagent (Invitrogen) per manufacturer’s instructions. RNA samples were quantitated using the Nanodrop 1000 spectrophotometer (Thermo Scientific, UK) and purity was ascertained by A260/280 ratio (>1.9).

### qPCR using Taqman® probes

Reverse Transcription: cDNA was synthesised by using Bioline Sensifast cDNA synthesis kit (Bio-65053). Taqman® oligonucleotide probes have been used in the qPCR experiments (Table 1) Real-time qPCR was done using the Bioline SensiFAST Probe Hi-ROX Kit (BIO-82002). The target genes included human β, γ globin. The qPCR reactions were performed in triplicates in 96- well optical plates (Microamp®, Applied Biosystems, N8010560).

The reactions were run on the Applied Biosystems Step-One Plus Real-Time PCR system machine. Results were analysed using the Life Technologies Step-One software.

### Determination of RNA expression levels by qPCR

Expression levels of human β and γ-globin chains (target genes) were assessed in the blood and bone marrow samples of treated and untreated mice at 12 weeks. The relative quantity of each target gene studied was calculated by the ΔΔCt method.

### Assessment of vector copy number by qPCR

Viral titers were quantified by determination of integrated viral copy number (VCN) in cell genomic DNA or unintegrated proviral vector genomes in the cytoplasm by a TaqMan® real-time qPCR method. The qPCR reactions were set-up in 96-well reaction plates (MicrAmpTM Optical; Applied Biosystems, USA) and analysed using Butler’s late reverse transcriptase (LRT) primer/probe set^105^.

The endogenous *titin* was used to determine absolute numbers of genomes present in each qPCR reaction using a primer/probe set. Titin, also called connectin, is a protein kinase involved in the structural assembly of microfilaments in muscle and during chromosome segregation and cell division in non-muscle cells. Standard curves were generated using serial dilutions (10^6^ copies to 10^1^ copies) of the GLOBE and the Titin plasmids (PGK plasmid)^106^ to determine copy number per cell[VCN per Cell = Number of Copies of GLOBEx2/Number of Copies of Titin].

### Primers and probes

The primers already described in the literature^19^ were used for the study. The oligonucleotides were custom-made from Eurofins MWG Operon, Germany (Table 1).

### Haematological indices

Peripheral blood was collected from anaesthetised mice using a heparinised syringe into BD micro trainer EDTA collection tubes (Fisher Scientific). Red blood cell counts and Hematocrit values were measured. Hb concentrations were determined by Hemocue Hb 201+ system. Full blood counts and Serum Protein Electrophoresis (SPE) analysis for all the samples were performed by Central Diagnostics facility at the University of Cambridge. Animal and Spleen weight was also measured using a high precision balance (Kern, UK).

### High-performance liquid chromatography analysis (HPLC)

Red cells (20 μl) were lysed in 500 μl 0.1M 2-mercaptoethanol/0.1M HCl and diluted to 1ml with 500 μl 50% aqueous acetonitrile. After centrifugation (13,000 rpm × 5 minutes), samples were transferred to HPLC autosampler vials and analyzed on a Grace Vydac 214TP C4 column (250 × 4.6 mm), using a gradient of solution A (water/acetonitrile/trifluoroacetic acid/heptafluorobutyric acid, 700:300:0.7:0.1) and solution B (water/acetonitrile/trifluoroacetic acid/heptafluorobutyric acid, 450:550:0.5:0.1). Proteins were detected using a Jasco MD-1510 detector and the area of globin chains quantified at 220 nm.

### Imaging

Pearl Stains and H&E stains were visualised using a Zeiss AxioScan Z1 Slide Scanner. The images were processed using Zeiss-lite software 2012.

### *In vivo* MRI

*In vivo* imaging, spleen volume, cardiac function and relaxometry were performed as described in L. Jackson *et al*.^23^, at 30 weeks of age using a 9.4T MR system (Agilent Technologies, Santa Clara, USA) equipped with 1000 mT/m gradient inserts and a 39mm volume resonator RF coil (RAPID Biomedical, Rimpar, Germany). A small animal physiological monitoring system (SA Instruments, Stony Brook, NY) was used to record the ECG trace, respiration rate, and internal temperature. Animals were anaesthetized with a mixture of isoflurane and oxygen with physiological measurements used to maintain the depth of anaesthesia^23^.

### Cardiac function

Cardiac function was assessed with a gradient echo cine MRI sequence with a temporal resolution of 5.2 ms, an in-plane spatial resolution of 117 μm and a slice thickness of 1 mm^23^. The left ventricular blood pool was segmented at systole and diastole using Segment v1.8 R0462^107^ and the corresponding volumes used to calculate left ventricular ejection fraction (EF), stroke volume (SV) and end systolic/diastolic volumes (ESV/EDV)^62^.

### Site integration studies

SI was performed by the Nationales Centrum für Tumorerkrankungen (NCT) Heidelberg, German Cancer Research Center (DKFZ), Department of Translational Oncology, Germany.

### Statistical analysis

All the haematological indices in the different groups (IUGT 32 Weeks, IUGT 12 Weeks, age-matched Humanised Non-Thalassemia Control, age-matched Uninjected Thalassemia Control group) were compared using one-way ANOVA with Bonferroni’s multiple comparison tests. For the qPCR data, statistical significance was determined by One Way ANOVA and Kruskal-Wallis test. To achieve a statistically meaningful result, for each group we aimed to have at least six mice pups in each group, born by more than three different dams, in order to have more than three independent biological replicates Graph Pad Prism Version 7.0a for MAC, Graph Pad Software, LA Jolla California, USA was used for statistical analyses and graph drawing.
