# Publisher Correction: In Utero Gene Therapy (IUGT) Using GLOBE Lentiviral Vector Phenotypically Corrects the Heterozygous Humanised Mouse Model and Its Progress Can Be Monitored Using MRI Techniques

**DOI:** 10.1038/s41598-019-55754-y

**Published:** 2019-12-24

**Authors:** Panicos Shangaris, Stavros P. Loukogeorgakis, Sindhu Subramaniam, Christina Flouri, Laurence H. Jackson, Wei Wang, Michael P. Blundell, Shanrun Liu, Simon Eaton, Nahla Bakhamis, Durrgah Latchumi Ramachandra, Panayiotis Maghsoudlou, Luca Urbani, Simon N. Waddington, Ayad Eddaoudi, Joy Archer, Michael N. Antoniou, Daniel J. Stuckey, Manfred Schmidt, Adrian J. Thrasher, Thomas M. Ryan, Paolo De Coppi, Anna L. David

**Affiliations:** 1 Institute for Women’s Health, University College London, 86-96 Chenies Mews, London, WC1E 6HX UK; 2 UCL Institute of Child Health, UCL, London, United Kingdom; 3 Biochemistry and Molecular Genetics, UAB, Birmingham, Alabama United States; 4 Department of Translational Oncology, National Centre for Tumour Diseases, Heidelberg, Germany; 5 Central Diagnostic Services, Queen’s Vet School Hospital, University of Cambridge, Cambridge, United Kingdom; 6 Centre for Advanced Biomedical Imaging, UCL, London, United Kingdom; 7 Wits/SAMRC Antiviral Gene Therapy Research Unit, Faculty of Health Sciences, University of the Witwatersrand, Johannesburg, South Africa; 8 Department of Medical and Molecular Genetics, KCL, London, United Kingdom

Correction to: *Scientific Reports* 10.1038/s41598-019-48078-4, published online 12 August 2019

In the original version of this Article, the author Paolo De Coppi was incorrectly indexed.

Additionally, Paolo De Coppi and Anna L. David were omitted as equally contributing senior authors.

These errors have now been corrected in the PDF and HTML versions of the Article.

